# Epidemiological survey and screening strategy for dengue virus in blood donors from Yunnan Province

**DOI:** 10.1186/s12879-021-05810-8

**Published:** 2021-01-22

**Authors:** Ling Li, Ying Li, Shaofang Lu, Jing Dong, Haixia Xu, Qian Zhang, Rong Weng, Yundi Yin, Rui He, Peng Fang, Hua Shi, Yinghan Yu, Ji Wu, Zhong Liu, John R. Hess

**Affiliations:** 1 Clinical Transfusion Research Center, Institute of Blood Transfusion, Chinese Academy of Medical Sciences (CAMS) and Peking Union Medical College, Chengdu, China; 2 Key Laboratory of Transfusion Adverse Reactions, CAMS, Chengdu, China; 3 Xishuangbanna Blood Center, Xishuangbanna, China; 4 Anhui Medical University, Hefei, China; 5 Department of Laboratory Medicine and Pathology, University of Washington School of Medicine, Seattle, USA

**Keywords:** Dengue, Transfusion-transmitted infection, Prevalence, Donor, Screening strategy

## Abstract

**Background:**

Dengue virus (DENV) infection is increasingly common in southern China and can be transmitted through blood transfusion but is not currently part of donor screening throughout the region. We assessed DENV prevalence among donors at the Xishuangbanna Blood Center, Yunnan, to support development of DENV screening strategies.

**Methods:**

Blood samples were collected randomly between June 2019 and August 2019. These were screened for anti-DENV IgG and IgM using enzyme-linked immunosorbent assay (ELISA). Then, all reactive samples and some randomly-chosen non-reactive samples were used to detect DENV RNAs using real-time polymerase-chain-reaction (RT-PCR) assays. After RT-PCR, samples were further tested for soluble nonstructural protein 1 (NS1) using the colloidal gold method. Donors demographics were also collected and assessed.

**Results:**

Over the study period, 2254 donor samples were collected and tested for anti-DENV IgG and IgM by ELISA. This revealed 598 anti-DENV IgG and/or IgM reactive samples, a serological prevalence of 26.53%. Of these, 26 were RT-PCR positive and/or NS1 positive. Significant differences in DENV prevalence were noted by occupation (*P = 0.001*), education (*P < 0.001*), and ethnicity (*P = 0.026*).

**Conclusion:**

The prevalence of DENV in Xishuangbanna Blood Center was higher than most other blood centers that have implemented DENV donor screening. Our study provides first-hand data about the prevalence of DENV and allows the development of a screening strategy for clinical use.

## Background

The rapid expansion of dengue fever is an evolving worldwide public health threat. The World Health Organization (WHO) estimates the overall prevalence of dengue at about 3.9 billion infections in mosquito-prone regions in 128 countries world-wide [1]. Roughly 96 million (25%) of these are identified clinically, with an annual incidence of about 390 million new cases [1]. In Africa, dengue is mostly unquantified, but recent outbreaks suggest that large parts of the continent are at increased risk [2].

Dengue virus enters the human body through the bite of Aedes mosquitoes, replicates in capillary endothelial cells and the mononuclear macrophage system, then enters the bloodstream as viremia. After infection, DENV replicates in the human body for 3 to 14 days before symptoms appear [3]. Since more than 75% of people infected with DENV are asymptomatic, the prevalence of people actively infected with dengue fever during outbreaks increases the risk of DENV infection among adult blood donors. At present, the dengue virus (DENV) is listed as a transfusion- transmitted disease (TTD) by the international transfusion association, the AABB [4]. To reduce the risk of transfusion-transmission of DENV, routine nucleic acid testing (NAT) of blood donor samples is recommended by several high prevalence countries and regions, such as Honduras [5].

China has recorded three outbreaks of dengue hemorrhagic fever. Most recently, this has included the large outbreak that spread to Guangdong Province in 2014, with 6024 confirmed cases and 6 deaths [6, 7]. This outbreak has sparked increasing concerns about DENV infection throughout the country, particularly with regard to blood safety. In China, DENV has not been included in routine screening of blood donors. However, with the rapidly increased population flow due to tourism, DENV has been spreading quickly, and serologic evidence of dengue infection among blood donors has been documented in Guangdong and Guangxi provinces [3, 8].

Given that no effective antiviral agents to treat dengue infection are available and that transmission of DENV by blood donors in mainland China is rising, investigation of the prevalence of DENV in high-risk areas is imperative. This includes analyzing DENV-positive blood donors’ demographic characteristics and evaluating whether wide-range DENV antibody screening is needed.

Xishuangbanna Dai Autonomous Prefecture in Yunnan Province is a region close to Laos and other Southeast Asian countries where dengue is prevalent and outbreaks occur every other year. Potentially infected blood donors in this area pose a threat to blood safety nationwide. However, prior to the study reported here, no data on dengue virus infection among blood donors in Yunnan Province has been collected or reported. In this study, we cooperated with Yunnan Xishuangbanna Blood Center to investigate the prevalence of dengue virus among blood donors, and, based on our findings, to develop a DENV screening strategy for high prevalence seasons and in high prevalence areas.

## Materials and methods

### Ethics statement

This study was approved by the Ethics Committee of the Institute of Blood Transfusion, Chinese Academy of Medical Sciences and Peking Union Medical College. Written informed consent was obtained from each study participant.

### Sample collection

This study was a collaboration between the Institute of Blood Transfusion (IBT) of the Chinese Academy of Medical Sciences and the Yunnan Xishuangbanna Blood Center. The samples were collected randomly from Xishuangbanna Blood Center donors from June through October 2019. Donor history questionnaires were obtained as a routine practice at the time of donation at the blood center. Procedures for sample collection and virus testing have been previously reported by our research group (Ling Li, 2019) [9].

### Testing algorithm

All collected samples were tested for anti-DENV IgG and IgM by ELISA.

A subset of these ELISA samples (including reactive and non-reactive samples) were then randomly selected to undergo screening for NAT and NS1. This step-wise approach was chosen because anti-DENV IgG and IgM can be tested by ELISA followed by NAT and NS1, and because the results of ELISA may be false positive. (See Discussion, below.)

### Anti-DENV IgG and IgM screening testing

All collected donor samples were screened for anti-DENV IgG and IgM, using Dengue virus IgG ELISA and Dengue virus IgM ELISA kits (ELISA, IBL International, Germany), respectively. Any reactive (IgG and/or IgM) plasma samples in collection tubes were then transferred to storage tubes (no EDTA) and stored at less than -20 °C. Some non-reactive (IgG and/or IgM) samples were selected randomly and an aliquot placed in NAT tubes with the remainder then transferred to storage tubes (no EDTA) and stored at less than -20 °C. These samples were then shipped on dry ice to the clinical transfusion center laboratory of IBT.

### DENV supplementary testing

**Fig. 1:**
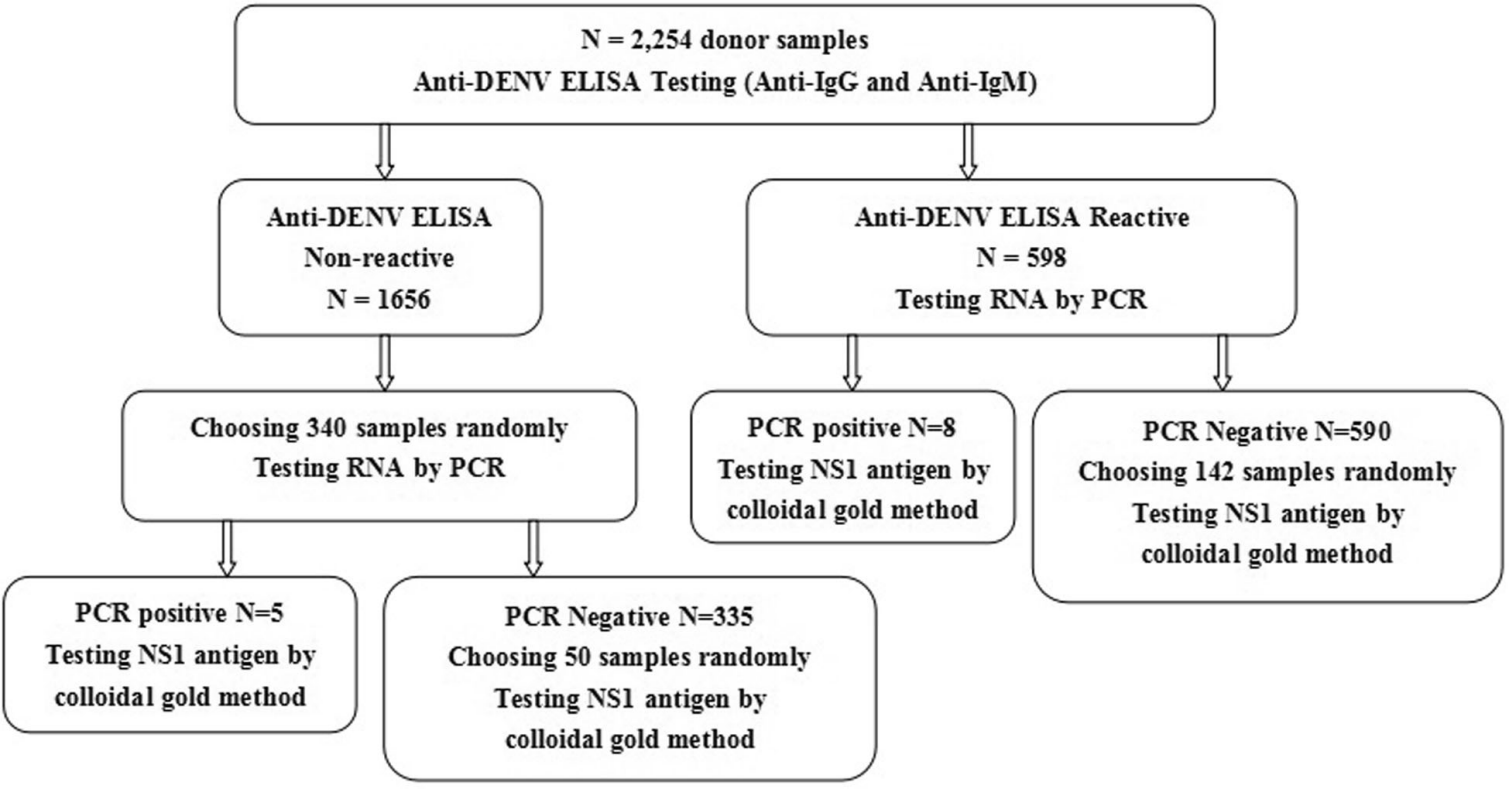
Testing algorithm

Further supplementary testing was performed on samples that were reactive on the DENV screening to confirm the presence of DENV, using RT-PCR (Shanghai ZJ Bio-Tech Co., Ltd., Shanghai,China) and NS1 testing (Colloidal gold method, Wondfo, Guangzhou, China). A blood donation sample was confirmed to be positive if either of the supplemental testing was also positive. Some non-reactive (IgG and/or IgM) samples were selected randomly and tested for RNA and NS1 antigen by PCR and the colloidal gold method. Figure 1 shows the algorithm of DENV testing.

Demographic characteristics of donors were also collected.

### Statistical analysis

Serological prevalence was calculated by the number of all reactive (IgG and/or IgM) samples divided by the number of total samples in each group. Pearson’s chi-square test was applied to evaluate the difference of serological prevalence between the donor sets (SPSS 17.0, IBM, Armounk, NY USA). Significance level α was set at *P* < 0.05.

## Results

### Summary of DENV screening

Table 1 shows the demographic characteristics and DENV testing results of all donors in Xishuangbanna Blood Center over the study period. Screening ELISA for anti-DENV IgG and IgM was completed 2254 donor samples. Of these samples, 598 (26.5%) were anti-DENV IgG and/or IgM reactive.

**Table 1:** The Demographic Characteristics and serological prevalence of DENV in each group

Characteristics	Only Anti-IgG reactive	Only Anti-IgM reactive	Anti-IgG And Anti-IgM reactive	Non-reactive	Total	Serological prevalence (%)	χ^2^ and *P* values
**Donors Status**							
First-Time	267	28	31	809	1135 (50.35%)	28.72	5.635, 0.018
Repeat	217	37	18	847	1119 (49.65%)	24.30	
**Gender**							
Male	331	39	29	1033	1432 (63.53%)	27.86	3.577, 0.059
Female	153	26	20	623	822 (36.47%)	24.21	
**Age Group**							
18 ~ 25	84	10	6	294	394 (17.48%)	25.38	9.337, 0.053
26 ~ 35	196	24	18	567	805 (35.71%)	29.57	
36 ~ 45	124	18	19	441	602 (26.71%)	26.74	
46 ~ 55	78	13	6	344	441 (19.57%)	22.00	
56 ~ 60	2	0	0	10	12 (0.53%)	16.67	
**Occupation**							
Farmers	76	9	12	188	285 (12.64%)	34.03	25.335, 0.001
Workers	34	3	3	116	156 (6.92%)	25.64	
Students	8	1	1	25	35 (1.55%)	28.57	
Soldiers	3	0	0	8	11 (0.49%)	27.27	
Teachers	4	0	0	34	38 (1.69%)	10.53	
Civil Servants	13	3	0	86	102 (4.52%)	15.69	
Medical staff	27	3	2	122	154 (6.83%)	13.01	
Employee	44	6	5	191	246 (10.91%)	22.36	
Others	275	40	26	886	1227 (54.44%)	27.79	
**Education**							
BHS^a^	239	30	33	639	941 (41.75%)	32.09	31.3, < 0.001
HSAD^b^	66	9	4	210	289 (12.82%)	27.33	
Bachelor	43	8	1	227	279 (12.38%)	18.64	
Master	3	0	0	10	13 (0.57%)	23.08	
Others	133	18	11	570	732 (32.48%)	22.13	
**Ethnicity**							
Han	240	35	27	885	1187 (52.66%)	25.44	14.36, 0.026
Bai	2	1	0	17	20 (0.89%)	15.00	
Miao	6	0	0	12	18 (0.80%)	33.33	
Dai	73	7	8	188	276 (12.24%)	31.88	
Buliang	14	1	1	18	34 (1.51%)	47.06	
Hani	70	11	3	248	332 (14.73%)	25.30	
Other	79	10	10	288	387 (17.17%)	25.58	
Total	484	65	49	1656	2254	26.53	

^a^*BHS* Below High School

^b^*HSAD* High School and Associate Degree

### Serological prevalence of DENV

As noted above, total serological prevalence was 26.5%. Serological prevalence was then calculated in various donor subgroups. Of the 2254 donors assessed, 1135 (50.35%) were first-time donors, and 1119 (49.65%) were repeat donors. Prevalence in first-time donors was 28.72% (326 out of 1135) and, in second time donors, 24.30% (272 out of 1119). Pearson’s chi-square testing suggested a significant difference in the prevalence of DENV in first-time donors and repeat donors (*P* = 0.018, χ^2^ = 5.635).

Numerically, prevalence was greater in male donors (27.86%, 399/1432) than females (24.21%, 199/822) and among those 26–35 years old than in other age groups. However, these differences were not statistically significant (males vs females, *P* = 0.059, χ^2^ = 3.577; by age group, *P* = 0.053, χ^2^ = 9.337).

Further analysis revealed substantial differences in the prevalence of DENV by occupation (*P* = 0.001, χ^2^ = 25.335), educational level (*P* < 0.0059, χ^2^ = 31.30) and ethnicity (*P* = 0.026, χ^2^ = 14.358). In particular, prevalence was highest among farmer donors (34.03%, 97/285), and there were significant differences in the prevalence of DENV between farmers and teachers (*P* = 0.003, χ2 = 8.622), civil servants (*P* < 0.001, χ2 = 12.233) and medical staff (*P* = 0.004, χ2 = 8.466).

The educational level of the largest proportion of donors (941 out of 2254, 41.75%) were Below High School (BHS), and this group also had the highest prevalence of DENV ELISA reactivity, 32.09% (302/941). Prevalence then decreased with increasing levels of education: high school and associate (HSAD), 27.33% (79/289); bachelor, 18.64% (52/279); master degrees, and others, 23.08% (3/13). Significant differences in prevalence were noted between those with BHS and bachelors’ degrees (*P* < 0.001, χ2 = 18.916) and BHS and HSAD (*P* < 0.014, χ2 = 6.052).

Among the various ethnic groups identified, prevalence was highest among those identifying as Buliang (47.06%, 16/34), Miao (33.33%, 6/18), and Dai (31.88%, 88/276); then Han (25.44%, 302/1187), Bai (15.00%, 3/20), Hani (25.30%, 84/332) and others (25.58%, 99/387). Significant differences were noted in prevalence among Buliang and Bai (*P* = 0.017, χ^2^ = 5.675), Hani (*P* = 0.007, χ^2^ = 7.353) and Han (*P* = 0.005, χ^2^ = 8.019).

### DENV RNA testing by PCR and NS1 antigen testing by colloidal gold method

#### ELISA reactive samples

Antibody-reactive donor samples were then tested for DENV RNA by PCR. Eight of the 598 samples were positive. These 8 PCR positive samples and 142 PCR negative samples (chosen randomly from 590 PCR-negative samples) were tested for NS1 antigen by the colloidal gold method. Six out of the eight PCR positive samples were NS1 positive, whereas two of the 142 PCR negative samples were NS1 positive, with six of the 142 samples being weakly positive. (That is, bands were seen with these samples but were weaker than the control band.)

#### ELISA non-reactive samples

We randomly chose 340 of the 1656 samples for RNA test by PCR. Five of these were RNA positive. Then these 5 RNA positive samples were tested for NS1 antigen by the colloidal gold method. Three out of the five were NS1 positive. We then chose 50 of the 335 RNA negative samples to test the NS1 antigen using the colloidal gold method. Four of the 50 samples were weakly positive; only one of the 50 was positive.

### Demographic characteristics

Table 2 provides detailed information on donors who were both ELISA reactive and PCR and/or NS1 positive. All 16 DENV screening ELISA reactive samples were PCR and/or NS1 positive. Of these eight female and eight male donors, ages ranged 21 to 44 years; ten were first-time donors. Educational levels for most (11 of16) were BHS or HSAD.

**Table 2:** The detailed information of donors who were ELISA reactive and PCR and/or NS1 Positive

Assigned Donor Number	Education	Occupation	Number of Blood Donation	Ethnicity	Blood Group	Results of Anti-IgG	Results of Anti-IgM	Results of NAT	Results of NS1
1	Bachelor	Medical staff	1	Dai	B	Reactive	Non-reactive	positive	Positive
2	High school	Others	2	Han	B	Reactive	Non-reactive	Positive	Positive
3	HSAD^b^	Employee	7	Dai	B	Reactive	Non-reactive	Positive	Positive
4	HSAD	Employee	1	Han	B	Reactive	Non-reactive	Positive	Positive
5	BHS^a^	Others	6	Han	B	Reactive	Non-reactive	Positive	Negative
6	BHS	Farmer	1	Dai	B	Reactive	Non-reactive	Positive	Positive
7	HSAD	Others	1	Dai	B	Reactive	Reactive	Positive	Positive
8	BHS	Others	1	Dai	O	Reactive	Reactive	Positive	Negative
9	HSAD	Others	1	Han	O	Reactive	Non-reactive	Negative	Positive
10	BHS	Others	2	Han	A	Reactive	Non-reactive	Negative	Positive
11	Bachelor	Employee	5	Han	O	Reactive	Non-reactive	Negative	WP^c^
12	Bachelor	Employee	1	Lagu	A	Reactive	Non-reactive	Negative	WP
13	BHS	Others	5	Dai	A	Reactive	Non-reactive	Negative	WP
14	High school	Others	1	Yi	A	Reactive	Non-reactive	Negative	WP
15	BHS	Others	1	Hani	B	Reactive	Non-reactive	Negative	WP
16	BHS	Others	1	Han	A	Reactive	Non-reactive	Negative	WP

^a^*BHS* Below High School

^b^*HSAD* High School and Associate Degree

^c^*WP* Weakly Positive

Table 3 provides detailed information on the 10 donors, five female, five male, who were ELISA non-reactive but PCR and/or NS1 positive. Ages ranged 26 to 48 years old. Five were first-time donors. Most (8 of 10) were in the lowest two educational groups.

**Table 3:** The detailed information of donors who were ELISA non-reactive but PCR and/or NS1 Positive

Assigned Donor Number	Education	Occupation	Number of Blood Donation	Ethnicity	Blood Group	Results of Anti-IgG	Results of Anti-IgM	Results of NAT	Results of NS1
1	Unkown	Medical staff	5	Dai	B	Non-reactive	Non-reactive	positive	positive
2	BHS^a^	Farmer	1	Dai	B	Non-reactive	Non-reactive	Positive	Positive
3	HSAD^b^	Others	2	Hani	B	Non-reactive	Non-reactive	Positive	Positive
4	HSAD	Farmer	5	Han	B	Non-reactive	Non-reactive	Positive	Negative
5	Bachelor	Others	1	Han	A	Non-reactive	Non-reactive	Positive	Negative
6	BHS	Farmer	2	Han	AB	Non-reactive	Non-reactive	Negative	WP^c^
7	BHS	Others	1	Lahu	B	Non-reactive	Non-reactive	Negative	WP
8	BHS	Others	7	Han	O	Non-reactive	Non-reactive	Negative	WP
9	BHS	Employee	1	Hani	O	Non-reactive	Non-reactive	Negative	WP
10	BHS	Others	1	Han	A	Non-reactive	Non-reactive	Negative	Positive

^a^*BHS* Below High School

^b^*HSAD* High School and Associate Degree

^c^*WP* Weakly Positive

## Discussion

In China, blood donation samples are routinely tested for HBV, HCV, HIV, and syphilis, but DENV has not been included in the routine donor testing. In the laboratory, testing for dengue is done using ELISA to screen for IgG/IgM, then confirmation using a colloidal gold method to test for NS1, and RT-PCR for specific RNA. Sensitivity of the NS1 test in the febrile phase of dengue can exceed 90% for primary infections, that is, those without prior infection, and antigenemia may persist for several days after the resolution of fever [10, 11].

In this study, we tested for evidence of DENV infection in blood samples from Xishuangbanna, a high-risk area of southwestern mainland China. Total serological prevalence was high at 26.53% (598 out of 2254) using anti-DENV-IgG and/or IgM ELISA. This prevalence may represent the highest serological prevalence ever reported [3, 7, 8]. Prevalence was significantly different between first-time and repeat donors, consistent with other pathogens such as HBV’s serological prevalence among blood donor categories [12, 13].

We also saw significant differences in the serological prevalence among occupational, educational, and ethnic groups. The serological prevalence for farmers and lower education-level donors was higher. We speculate that this occupation-related phenotype has something to do with the donors’ living environment where the risk of DENV transmission was high. In fact, DENV is mainly transmitted by the *Aedes aegypti*s, often found in residential areas, containers (such as tanks, basins, discarded tires, etc.), plant containers (such as bamboo tubes, tree holes, etc.), and stone pools. It is most likely that residential areas where farmers live are more suitable for *Aedes aegypti*.

A few donors were RNA and/or NS1 positive among IgG and/or IgM reactive donors. This may be due to the overlapping of various markers’ time of appearance in plasma after DENV infection [14]. RNA and NS1 of DENV are the earliest markers appearing in plasma—either can appear within the first 24 h in which patients become symptomatic—and lasting 5 to 7 days [14]. In contrast, IgG (secondary infection) and IgM appear in plasma after day 4 and last 6 days [14]. This result shows that NS1 can be detected at the same time as viral RNA. However, the detection of NS1 might be limited during secondary infection because of pre-induced adaptive immunity. Generally, the window period of nucleic acid detection is shorter than that of antibody detection. However, for dengue virus, the risk of missing the true positive case is high if there is only a nucleic acid test to screen for dengue virus prevalence because the nucleic acid disappears quickly from plasma: 590 out of 598 (98.66%) ELISA reactive samples were PCR negative. Our findings, together with others [15], indicate that to increase the likelihood of DENV detection and reduce the risk of blood transfusion-transmitted DENV, it may be essential to perform a joint test for of DENV RNA, NS1, and IgG/IgM antibodies.

Table 2 and Table 3 show that the donors who were RNA and/or NS1 positive were mostly farmers or other occupations with higher exposure to standing water. Efforts at mosquito control in the surrounding environment will help reduce DENV infection, but multilayer testing will be necessary to decrease blood transfusion-transmitted DENV.

## Conclusion

The prevalence rate of DENV in Xishuangbanna Blood Center is higher than most other blood centers that have implemented DENV donor screening. In order to reduce the risk of transfusion-transmitted DENV, it may be necessary to propose a DENV screening strategy based on these findings in high prevalence seasons and high prevalence areas.

## Data Availability

The datasets used and/or analysed during the current study are available from the corresponding author on reasonable request.

## Abbreviations

TTD: Transfusion-Transmitted disease
DENV: Dengue virus
HBsAg: Hepatitis B surface Antigen
HCV: Hepatitis C
HIV: Human immunodeficiency virus
NAT: Nucleic Acid Tests
ELISA: Enzyme-Linked Immuno Sorbent Assay
